# Green Tea Polyphenols Reduced Fat Deposits in High Fat-Fed Rats via erk1/2-PPARγ-Adiponectin Pathway

**DOI:** 10.1371/journal.pone.0053796

**Published:** 2013-01-15

**Authors:** Chong Tian, Xiaolei Ye, Rui Zhang, Jia Long, Weiye Ren, Shibin Ding, Dan Liao, Xin Jin, Hongmei Wu, Shunqin Xu, Chenjiang Ying

**Affiliations:** 1 Department of Nutrition and Food Hygiene, School of Public Health, Tongji Medical College, Huazhong University of Science and Technology, Wuhan, People’s Republic of China; 2 Ministry of Education Key Lab of Environment and Health, School of Public Health, Tongji Medical College, Huazhong University of Science and Technology, Wuhan, People’s Republic of China; 3 Department of Public Health, WenZhou Medical College, WenZhou, People’s Republic of China; Universidad Miguel Hernández de Elche, Spain

## Abstract

**Objective:**

Hypoadiponectinemia contributes to the development of obesity and related disorders such as diabetes, hyperlipidemia, and cardiovascular diseases. In this study we investigated the effects of green tea polyphenols (GTPs) on adiponectin levels and fat deposits in high fat (HF) fed rats, the mechanism of signaling pathway was explored as well.

**Methods and Results:**

Male Wistar rats were fed with high-fat diet. GTPs (0.8, 1.6, 3.2 g/L) were administered via drinking water. Serum adiponectin and insulin were measured by ELISA, mRNA levels of adiponectin and PPARγ in visceral adipose tissue (VAT) were determined by Real-time PCR, protein levels of PPARγ, phospho (p) - PPARγ, extracellular signal regulated kinase (erk) 1/2 and p-erk1/2 in VAT were determined by western blot. GTPs treatment attenuated the VAT accumulation, hypoadiponectinemia and the decreased mRNA level of adiponectin in VAT induced by HF. Decreased expression and increased phosphorylation of PPARγ (the master regulator of adiponectin), and increased activation of erk1/2 were observed in HF group, and these effects could be alleviated by GTPs treatment. To explore the underlying mechanism, VAT was cultured in DMEM with high glucose to mimic the hyperglycemia condition *in vitro*. Similar to the results of *in vivo* study, decreased adiponectin levels, decreased expression and increased phosphorylation of PPARγ, and elevated erk1/2 phosphorylation in cultured VAT were observed. These effects could be ameliorated by co-treatment with GTPs or PD98059 (a selective inhibitor of erk1/2).

**Conclusion:**

GTPs reduced fat deposit, ameliorated hypoadiponectinemia in HF-fed rats, and relieved high glucose-induced adiponectin decrease in VAT *in vitro*. The signaling pathway analysis indicated that PPARγ regulation mediated via erk1/2 pathway was involved.

## Introduction

With a tremendous increase in China, the prevalence of obesity has been rising all over the world in the last few decades [1]. Obesity is a vital risk factor for a number of chronic diseases, such as diabetes, vascular diseases, and cancers. Therefore, prevention of obesity becomes crucial in public health. The medical and surgical methods have been used to treat obesity, however, the adverse effects of these treatments are obvious [2], [3]. Usage of dietary agents to prevent or treat obesity could be a safer solution to the problem. Epidemiological and laboratory studies showed that green tea possesses significant anti-obesity, anti-diabetes, and cardio-protective properties [4], 5,6. Epigallocatechin 3-gallate (EGCG), the major component of catechines in green tea, was demonstrated to reduce body weight gain in animal models of obesity [7], [8]. Human intervention studies showed that catechins, the bioactive ingredients of green tea polyphenols (GTPs), decreased body weight and affected markers of obesity in healthy, obese and diabetic conditions [9]–[11]. These data indicated that tea polyphenols are potentially powerful agents in the prevention of obesity. It was suggested that tea decreases body weight gain by reducing lipid and carbohydrate absorption, increasing lipid and carbohydrate utilization, and lessening the lipogenesis, but the mechanism of tea’s anti-obesity effects is still inconclusive.

Adiponectin (also referred to as GBP-28, apM1, AdipoQ and Acrp30) is an adipocytokine exclusively secreted by adipose tissue into the blood stream. Adiponectin counts for approximately 0.01% of the total plasma proteins. Hypoadiponectinemia, which refers to a low circulating level of adiponectin, was documented in obesity and its related diseases including insulin resistance, hyperglycemia and cardiovascular diseases [12]–[14]. Accumulating evidence showed that hypoadiponectinemia played a key role in the pathogenesis of obesity and related diseases [15]–[17]. Furthermore, adiponectin administration to obese or diabetic mice can reduce body weight and blood glucose levels while enhancing insulin sensitivity [18]–[20]. Based on these data, adiponectin was conceived to be a novel therapy target for obese and insulin resistance [21]. Elevation of adiponectin level by EGCG was documented in non-obese spontaneous diabetic rats and spontaneous hypertension rats [22], [23]. Based on the above research, we hypothesized that GTPs might regulate the adiponectin levels in HF fed rats, through which GTPs exert their preventive effects on obesity and related diseases.

Adiponectin expression is regulated by factors such as peroxisome proliferator-activated receptor (PPAR) γ, CCAAT-enhancer-binding protein (C/EBP) α, Kruppel-like factor 7 (KLF7), and sterol regulatory element binding protein-1c (SREBP-1c), Among these factors, PPARγ is recognized as the master regulator of adiponectin gene transcription. PPARγ binds directly to a functional PPAR-responsive element (PPRE) in adiponectin promoter, and increases the transcription of adiponectin gene [24]. Thiazolidinediones (TZDs), agonists of PPARγ, stimulated gene expression and increased the plasma level of adiponectin in obese mice and obese people with insulin resistance [25]. Dominant negative mutant in PPARγ gene inhibited circulating adiponectin by 5 times [26]. Although the results were inconsistent, PPARγ expression was reported to change in obese or diabetic conditions. Park et al. and Inoue et al. reported PPARγ expression increased in skeletal muscle, liver or adipose tissue of obesity or diabetic subjects [27], [28]. Another study showed that long term over-feeding induced obesity and decreased PPARγ expressions in skeletal muscle and VAT [29]. The mechanism of these contradictory results was not fully understood. Evidence showed erk1/2 activation was involved in regulation of PPARγ expressions in different tissues [30], [31]. A MAP kinase site, which is confirmed to be phosphorylated by MAPKs, was identified on the NH2-terminal domain of PPARγ, and phosphorylation on the site would repress the transcription activity of PPARγ then decrease the adiponectin level [32]. Increased phosphorylation of PPARγ was observed in obesity and insulin-resistant conditions, and the involvement of increased activation of erk1/2 was proposed [33]. Regulating effects of GTPs on the expression of PPARγ were reported though the results were not consistent [34]. Additionally, our previous study demonstrated GTPs would affect the MAPK signal pathways [35]. Based on these facts, we hypothesized that GTPs may regulate adiponectin levels via PPARγ by modulating erk1/2 activations.

In this study, male Wistar rats were fed on HF diet, and the effects of GTPs on body weight gain and adiponectin levels were observed. The signaling pathway mechanism was explored along with the regulatory roles of PPARγ and erk1/2.

## Materials and Methods

### 1. Ethics Statement

This study was carried out in strict accordance with the guidelines and authorization for the use of laboratory animals. The protocol was approved by the Committee on the Ethics of Animal Experiments of the Huazhong University of Science and Technology (Permit number: S249). All efforts were made to minimize suffering.

### 2. Reagents and Materials

Anti-β actin was from Santa Cruz Biotechnology, Inc. (Santa Cruz. CA. USA). Anti-erk1/2 and p-erk1/2 antibodies were purchased from Cell Signaling Technology (Billerica, MA, USA). Anti-PPARγ antibody was purchased from Abcam (Cambridge, MA, USA); GTPs (purity >98%) were purchased from Fuzhou Rimian Inc. (Fuzhou, Fujian, China); TRIZOL was from Invitrogen Inc. (Carlsbad, CA, USA) and Real time quantitative PCR kit was purchased from TAKARA Bio Inc. (Otsu, Shiga, Japan); ELISA kits for adiponectin and insulin were purchased from R&D Systems. (MN, USA). All other chemicals were of the highest grade commercially available.

### 3. Animals

After one week’s acclimation, thirty male Wistar rats, weighting 40–60 g, were randomly divided into 5 groups. The control group was fed on standard chow; the other 4 groups were fed with modified HF chow containing 60% (w/w) standard chow, 12% lard, 12% sugar, 6% peanuts powder, 8% yolk powder, and 1% milk powder. Since the 4th week, 3 of the 4 HF groups started to drink water containing different concentrations of GTPs (0.8, 1.6, 3.2 g/L). At the end of the 26^th^ week, all animals were sacrificed, tissues were snap frozen with liquid nitrogen then stored at −80°C freezer. Blood biochemical indexes, adiponectin levels and insulin levels were tested. This study was carried out in strict accordance with the guidelines and authorization for the use of laboratory animals. The protocol was approved by the Committee on the Ethics of Animal Experiments of the Huazhong University of Science and Technology. All efforts were made to minimize suffering.

### 4. VATs Culture

Male Wistar rats were sacrificed and VATs were collected under asepsis condition. One hundred fifty mg VAT was maintained at 37°C in 5% CO2. VATs cultured in DMEM with high glucose were treated without GTPs, with GTPs (4 µg/ml) for 48 hrs, or treated with PD98059 for 1 hour, respectively. VATs incubated with 5.5 mmol/L glucose in medium were used as control.

### 5. Quantitative Real-time PCR

Following the manufacturer’s instructions, total RNA was extracted from fat tissue using TRIZOL then quantified by UV spectrophotometry. Samples with the A260/280 ratio lies between 1.8∼2.0 were used. Reverse transcription reaction (RT) was performed with 1 µg total RNA from each sample using random primers. Real time PCR analysis was carried out using qPCR SYBR Green mix with the following parameters: 1 cycle, 95°C, 5 s; 40 cycles, 95°C 10 s; 57°C, 30 s. Changes of gene expression were determined by the comparative Ct method with GADPH as reference. The primers used in the PCR were as follows: GADPH: glyceraldehyde-3-phosphate dehydrogenase (GAPDH) (BC059110) sense: CAG TGC CAG CCT CGT CTC AT, antisense: AGG GGC CAT CCA CAG TCT TC; Adiponectin (NM144744) sense: GGT GAC CAG GAG ATG CT, antisense: TAC GCT GAA TGC TGA GTG ATA; PPARγ (NM001145366) sense: TCA GGT TTG GGC GAA TG, antisense: TTT GGT CAG CGG GAA GG.

### 6. Enzyme-linked Immunosorbent Assay

To assess adiponectin secretion and circulating insulin, the blood of the rats was collected and serum was separated by centrifugation, and the culture supernatant of fat explants was harvested and centrifuged to remove impurities. Adiponectin and insulin concentrations were measured using an enzyme-linked immunosorbent assay according to the manufacturer’s instruction. Measurements were performed in six replicates. Results were presented as ng/mL.

### 7. Electrophoresis and Immunoblotting

Fat tissue were homogenized and then lysed in the extraction buffer containing 50 mmol/L Tris/HCl (pH 8.0), 150 mmol/L NaCl, 1% Nonidet-P40, 1% sodium deoxycholate, 0.1% sodium dodecyl sulfate (SDS), 0.1 mmol/L DTT, 0.05 mmol/L PMSF, 0.002 mg/ml aprotinin, 0.002 mg/ml leupeptin, and 1 mmol/L NaVO3 [36]. The protein concentration was quantified with BIO-RAD DC Protein Assay Reagent (Bio-Rad, Hercules, CA, USA). Sodium dodecyl sulfate-polyacrylamide gel electrophoresis and immunological blotting was performed according to the method of Amersham Biosciences. Protein expression was visualized with a chemiluminescent detection system (Syngen, Cambridge, UK) and analyzed by Gel Pro 3.0 software (Biometra, Goettingen, Germany).

### 8. Statistical Analysis

All quantitative data are presented as Mean ± S.E. Data were compared by ANOVA-SNK or Dunnett’s T3 test. Differences were considered significant when *P*<0.05.

## Results

### 1. GTPs Reduced Body Weight Gain and VAT Coefficient without Affecting the Food Intake

**Figure 1:**
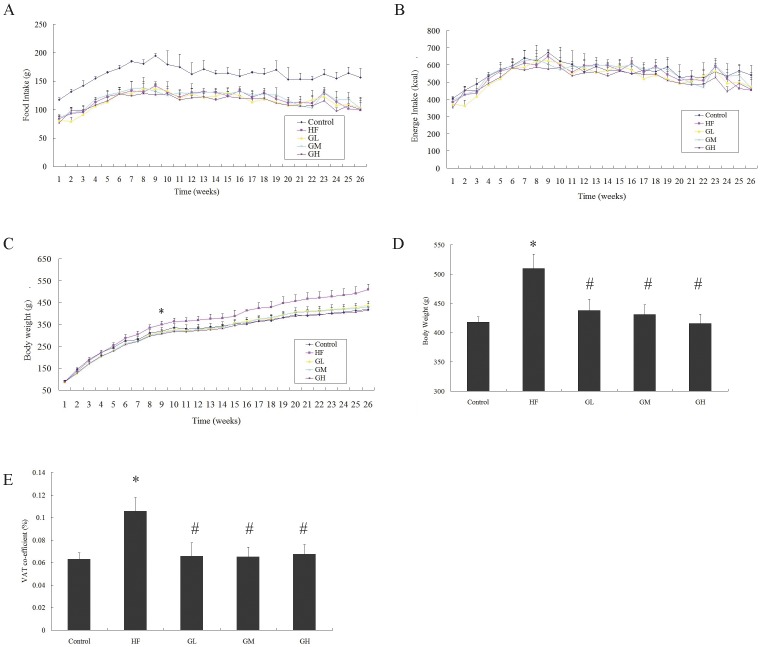
GTPs reduced body weight gain and VAT coefficient induced by HF diet without affecting the energy intake. GTPs treatment did not affect the energy intake in compare with the HF group, although the food intake of the control group is higher than other groups(A), no significant difference was observed in energy intake (B). Difference in body weight started from the 9^th^ week (C). The body weight was obviously higher in the HF group in compare with all other groups (D); VAT coefficient of the HF group was obviously higher than the control group, and GTPs treatment alleviated the effect (E). (* *P*<0.05 vs. the control; # *P*<0.05 vs. the HF group). Data is expressed as the mean ± SEM (N = 6).

As shown in fig. 1, GTPs treatment did not affect the energy intake in comparison to the HF group, although the food intake of the control group was higher than other groups, no significant difference was observed in energy intake due to the different energy densities of normal and HF diet (A, B). Difference in body weight gain started from the 9^th^ week (C). At the end of the experiment, the body weight and VAT coefficient were obviously higher in the HF group; the effects were ameliorated by GTPs treatment (D,E). GTPs treatment also reduced the blood glucose and HOMA-IR index and reversed the lipid profile change in HF fed rats (Table 1). However, GTPs treatment did not affect the circulating insulin level compared to the HF group.

**Table 1:** GTPs downregulated the blood glucose and improved the lipid profile in HF fed rats.

	Control	HF	GL	GM	GH
Blood Glucose(mmol/L)	5.55±0.48	6.42±0.16*	5.96±0.40*#	5.93±0.41*#	5.40±0.46*#
Insulin (ng/mL)	11.47±0.81	14.73±0.58*	13.97±1.11*	14.53±1.70*	14.13±0.88*
Homa-IR	2.71±0.07	4.16±0.10*	3.59±0.19*#	3.58±0.24*#	3.27±0.11*#
TC(mmol/L)	1.06±0.10	1.46±0.04*	1.38±0.13*	1.06±0.16#	0.73±0.12*#
TG(mmol/L)	0.51±0.07	0.73±0.14*	0.64±0.10*	0.65±0.10*	0.43±0.12#
LDL-C/HDL-C	0.53±0.02	1.86±0.29*	1.48±0.08*#	0.65±0.05#	0.31±0.08*#

* *P*<0.05 vs. the control;

# *P*<0.05 vs. the HF group.

### 2. GTPs Relieved the Down-expression of Adiponectin in VAT and Serum Induced by HF Diet

**Figure 2:**
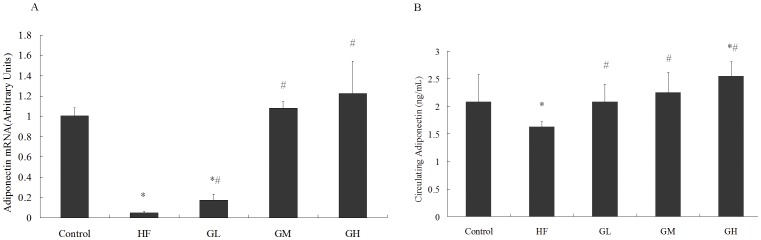
GTPs alleviates the decrease of adiponectin expression in fat tissue and serum induced by HF diet. Fig. 2 A showed the mRNA level of adiponectin in adipose tissue, the result is presented in arbitrary units using GADPH as reference. Fig. 2 B presented the levels of serum adiponectin. The HF group exhibited significantly reduced mRNA and circulating levels of adiponectin, the decreased expressions were attenuated by GTPs treatment at different concentrations (GL 0.8 g/L, GM 1.6 g/L, GH 3.2 g/L.) (* P<0.05 vs. the control; # P<.05 vs. the HF group). Data is expressed as Mean ± SEM (N = 6).

To assess the adiponectin expression and secretion, adiponectin mRNA in VAT was tested by qRT-PCR, and serum adiponectin was tested by ELISA. Fig. 2 demonstrated that HF fed rats exhibited lower expressions of adiponectin at transcriptional and phenotypic levels, while treatment of GTPs alleviated the adiponectin-reducing effect of HF diet.

### 3. GTPs Attenuated the Down-expression of PPARγ, the Increased Phosphorylation of PPARγ, and the Increased Phosphorylation of erk1/2 in VAT Induced by HF Diet

**Figure 3:**
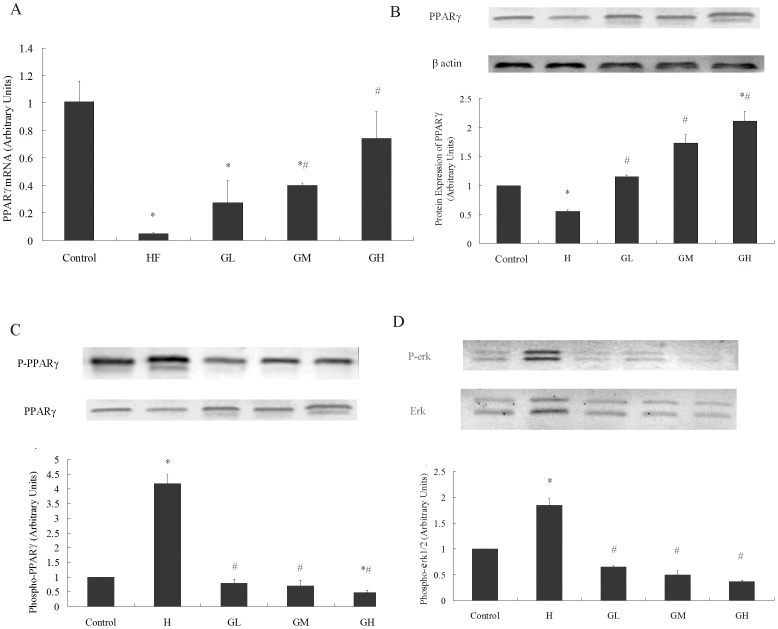
GTPs attenuated the phosphorylation, decreased expression of PPARγ and erk1/2 activation in fat tissue induced by HF diet. PPARγ mRNA level was calculated with GADPH as reference. Protein expression and phosphorylation of PPARγ and erk1/2 were tested by western blot; the results were presented in arbitrary units using beta-actin, PPARγ and erk1/2 as references, respectively. The value of the control group was considered as 1.00. HF down-regulated the mRNA (A, N = 6) and protein expression (B, N = 3) of PPARγ while up-regulated the phosphorylation of PPARγ (C, N = 3) and erk1/2 (D, N = 3), the effects could be ameliorated by GTPs treatment. (* P<0.05 vs. the control; # P<0.05 vs. the HF group). Data is expressed as Mean ± SEM.

Researches demonstrated that adiponectin is a downstream target gene of PPARγ [13] while p-PPARγ preserves an inhibitory effect on adiponectin expression [20]. The mRNA of PPARγ and protein expression of PPARγ and p-PPARγ were tested by qRT-PCR and Western Blot, respectively. As shown in Fig. 3, both the mRNA expression (A) and protein expression (B) of PPARγ decreased in HF group while phosphorylation of PPARγ (C) increased in HF fed group, and these changes were attenuated by GTPs treatment. Increased phosphorylation of erk1/2 induced by HF diet was also ameliorated by GTPs treatment (D).

### 4. GTPs or Selective Inhibitor of erk1/2 Relieved High Glucose-induced Adiponectin Decrease

**Figure 4:**
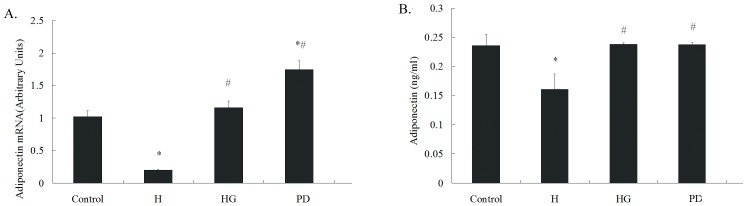
GTPs and selective inhibitor of erk1/2 alleviated high glucose-induced adiponectin decrease. One hundred fifty mg VAT were cultured in DMEM with high glucose (33 mmol/L) and cotreated with GTPs (4 µg/ml) for 48 hrs or pretreated with PD98059 for 1 hr. The supernatant of cell culture medium was collected for ELISA of secreted adiponectin. (A) The mRNA level of adiponectin, comparative Ct method with GADPH as reference was adopted. (B) The secreted adiponectin in the supernatant of culture medium is in ng/mL. High glucose incubation (H) down-regulated the mRNA expression and secretion of adiponectin, the effects could be attenuated by GTPs treatment (GH) or PD98059(PD). (* P<0.05 vs. the control; # P<0.05 vs. the HG group). Data is expressed as Mean ± SEM (N = 6).

Fig. 4 showed adiponectin’s mRNA level in cultured VAT (A) and its secretion in culture supernatant (B). High glucose down-regulated the mRNA expression in VATs and the secreted amount in culture medium. Both GTPs co-incubation and treatment with PD98059 alleviated the adiponectin-reducing effect of high glucose.

### 5. GTPs and Selective Inhibitor of erk1/2 Relieved the Down-expression of PPARγ and the Increased Phosphorylation of PPARγ and erk1/2 in Cultured VAT

**Figure 5:**
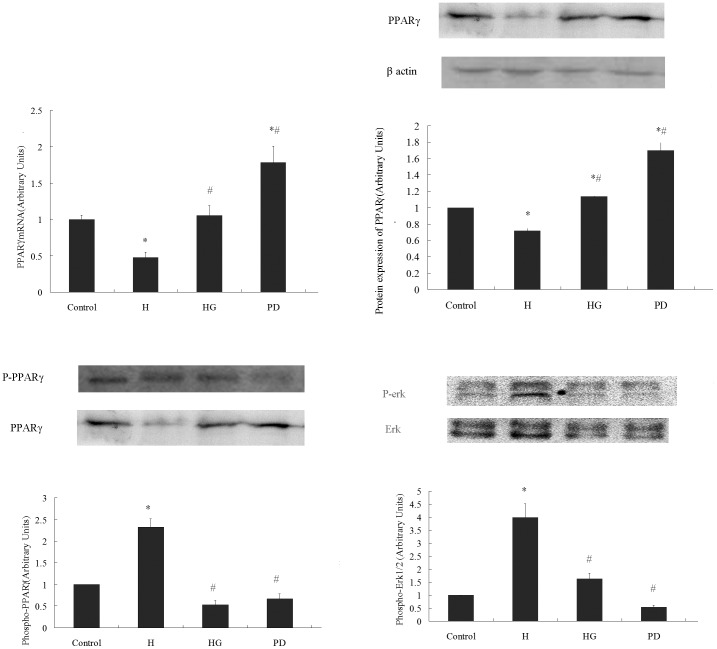
Inhibition of erk1/2 and GTPs treatment attenuated the phosphorylation and down-expression of PPARγ and erk1/2 activation in cultured VAT under high glucose condition. The cultured VAT explants were treated with GTPs and PD98059, respectively. High glucose incubation down-regulated the PPARγ mRNA (A, N = 6) and protein expression (B, N = 3) while up-regulated the phosphorylation of PPARγ (C, N = 3) and phosphorylation of erk1/2 (D, N = 3), all the above effects could be attenuated by GTPs or PD98059. (*P<0.05 vs. the control; # P<0.05 vs. the HG group). Data is expressed as Mean ± SEM.

As shown in Fig. 5, both mRNA (A) and protein expressions (B) were down-regulated by high glucose incubation in cultured VATs. GTPs treatment diminished the PPARγ-reducing effect of high glucose incubation, and inhibition of erk1/2 by PD98059 exerted similar effects. Both GTPs and PD98059 treatments attenuated the elevated phosphorylation of PPARγ (C) and erk1/2 (D) in high glucose incubated group.

## Discussion

The present work demonstrated that GTPs alleviated VAT deposit and hypoadiponectinemia in HF fed rats. Meanwhile, GTPs up-regulated the expression while down-regulating the phosphorylation of PPARγ, the principal regulator of adiponectin. GTPs also inhibited the over-activation of erk1/2 induced by HF diet. Similar results were observed in high glucose incubated VAT, co-treated with GTPs *in vitro.* Like being treated with GTPs, selective inhibition of erk1/2 alleviated the down-expression of adiponectin, down-regulated phosphorylation of PPARγ, and up-regulated the expression of PPARγ induced by high glucose incubation.

Adiponectin was demonstrated to be adversely associated with obesity, insulin resistance, cardiovascular diseases, and obesity related fatty liver disease [37], [38]. The production of adiponectin was reported to be related to visceral fat deposits [39]. Hypoadiponectinemia was observed in obese humans [40] and obese animal models in the present study, while increased adiponectin levels was observed after weight loss [41]. Genetic studies showed that adiponectin polymorphism, SNPs 45T to G and 276G to T are related to obesity in humans [42] and the G/G genotype for SNP276 was associated with lower serum adiponectin levels and waist-to-hip ratio [43], novel genetic determinents of adiponectin levels were identified in 2012 and the identified loci were proved to impact upon metabolic diseases [44]. Furthermore, intravenous or intra-cerebro-ventricular administration of adiponectin decreased body weight [2], [45]. Diet composition and exercise, which are closely related to body weight, were showed to affect plasma adiponectin levels. Reports demonstrated that HF diet decreased adiponectin levels [46], [47], which is consistent with the present study. While low fat, high carbohydrate diet [48], diets low in glycemic load and high in fiber [49], and food restriction [50], [51] increased adiponectin levels. Exercise was demonstrated to increase adiponectin levels in humans and animals [52], [53]. These reports suggested that food composition or exercise affect body weight via regulating adiponectin. Therefore, means to increase adiponectin level was conceived to be a novel therapy strategy for obesity and related diseases [2]. Similar to adiponectin, GTPs consumption was reported be associated with obesity, metabolic syndrome, type 2 diabetes and cardiovascular diseases [2]. In this study, GTPs treatment alleviated VATs increase and blood glucose elevation, and improved the insulin sensitivity and lipid profile in the HF fed rats. At the same time, GTPs treatment attenuated the decrease of adiponectin induced by HF or high glucose, which was also obeserved in another research using tea extracts [54]. From this point, regulation of adiponectin should be related to the mechanism by which GTPs exert anti-obesity, anti-diabetic and cardiovascular protective effects. However, further studies to investigate the effects of GTPs on adiponectin knockout mice would help consolidating the conclusion.

Gene expression of adiponectin is mainly regulated by nuclear transcriptor named PPARγ. PPARγ binds with PPRE element in the adiponectin gene and stimulates the transcription [13]. Research demonstrated PPARγ agonists would increase the circulating adiponectin in a metabolic syndrome rat model [55], and an epidemiological study proved that PPARγ gene polymorphism would affect the serum adiponectin levels [56]. PPARγ expression reduction was observed in obesity subjects [57], [58]. In our experiments, decreased mRNA and protein expressions of PPARγ and adiponectin were observed in HF fed rats and high glucose incubated VATs, and these effects could be attenuated by GTPs treatment. The transcription activity of PPARγ was demonstrated to be affected by several factors, including phosphorylation or sumoylation of the receptor [59], [60] and recruitment of different cofactors [61], among which phosphorylation of PPARγ is investigated most. Phosphoryltion of PPARγ resulted in decreased PPARγ activation followed by down-regulation of adiponectin gene [2]. Genetic techniques of inhibiting PPARγ’s phosphorylation could improve insulin resistance and increase adiponectin level [62]. Along with the decrease of adiponectin expression, increased phosphorylation of PPARγ was observed *in vitro* and *in vivo* in the present study, and all these effects could be attenuated by GTPs treatment. Meanwhile, phosphorylation of PPAR gamma would prime PPARγ for poly-ubiquitination and proteasomal degradation, increase the sumoylation of K77/K107 in a lysine motif IKVE directly adjacent to S82/112, and then synergistically repress PPARγ transactivation [63]. Activation of erk1/2, which was documented in adipocytes of obese rodents and humans [64], was confirmed to induce phosphorylation of PPARγ [65]. In the present study, HF diet and high glucose incubation elevated erk1/2 activation and phosphorylation of PPARγ, which was consistent with the study conducted by Hosooka et al [66]. Treatment with GTPs inhibited the activation of erk1/2, and alleviated the decreased PPARγ expression and increased PPARγ phosphorylation induced by HF diet *in vivo* or by high glucose incubation *in vitro*. Selective inhibition of erk1/2 by PD98059 exerts same effects as GTPs treatment. These results suggested that GTPs increased PPARγ expression and inhibited PPARγ phosphorylation by down-regulating erk1/2 in HF fed rats or under high glucose condition. However, the regulatory roles of erk1/2 activation played on PPARγ expression remain uncertain. Erk1/2 might directly down- regulate the PPARγ expression, as well as reduce PPARγ expression by triggering the phosphorylation of PPARγ.

### Conclusion

GTPs prevent HF-induced obesity by up-regulating adiponectin levels. The underlying mechanisms may include the inhibition of erk1/2 activation, alleviation of PPARγ phosphorylation, and increase of the PPARγ expression. Further research to track the PPARγ and adiponectin level along with the pathogenesis of obesity would lead to better understanding of the mechanism.
