# Correction: Green Tea Polyphenols Reduced Fat Deposits in High Fat-Fed Rats via erk1/2-PPARγ-Adiponectin Pathway

**DOI:** 10.1371/annotation/83355f31-f12d-4b8e-9310-b60d11e37482

**Published:** 2013-09-20

**Authors:** Chong Tian, Xiaolei Ye, Rui Zhang, Jia Long, Weiye Ren, Shibin Ding, Dan Liao, Xin Jin, Hongmei Wu, Shunqin Xu, Chenjiang Ying

In the Materials and Methods section, the last sentence of subsection "6. Enzyme-linked Immunosorbent Assay" should read "The levels of insulin were presented by ng/mL, and the levels of adiponectin were presented by μg/mL."

In Figures 2 and 4, the term “Adiponectin (ng/mL)” should be “Adiponectin (μg /mL).”

In the legend of Figure 4, “ng/mL”should be “μg/mL." 

